# Optimizing Ultrasonography of the Nasal Cartilage for Rhinoplasty: Techniques and Challenges

**DOI:** 10.1111/jocd.16543

**Published:** 2024-09-20

**Authors:** Wei‐Ting Wu, Ke‐Vin Chang, Levent Özçakar

**Affiliations:** ^1^ Department of Physical Medicine and Rehabilitation National Taiwan University Hospital Taipei Taiwan; ^2^ Department of Physical Medicine and Rehabilitation National Taiwan University College of Medicine Taipei Taiwan; ^3^ Center for Regional Anesthesia and Pain Medicine, Wang‐Fang Hospital Taipei Medical University Taipei Taiwan; ^4^ Department of Physical and Rehabilitation Medicine Hacettepe University Medical School Ankara Turkey

**Keywords:** aesthetic medicine, nose, septal cartilage, surgery, ultrasound

To the Editor,

The nasal cartilage can be categorized into two main types based on its anatomy: lateral and septal cartilages. The former include the upper and lower lateral (alar) cartilages. The upper lateral cartilages are paired structures that extend from the nasal bones downward and outward, forming the sides of the upper part of the nose. The lower lateral cartilages, also paired, are located below the upper lateral cartilages. They play a crucial role in shaping the nostrils and the tip of the nose, and are typically divided into medial, intermediate, and lateral crura—based on their positions and roles in shaping the nasal tip and nostrils [1].

The septal cartilage is a single, central cartilage that runs down the middle of the nose, separating the two nasal cavities. It provides structural support and helps maintain the shape of the nose. During rhinoplasty, surgeons may use a small piece of septal cartilage to reshape the nose [2]. This often involves harvesting a spreader graft from the nasal septum. For revision surgeries, it is essential to determine if the remaining septal cartilage is thick enough for another harvest. Preoperative imaging of the nasal septum cartilage is therefore crucial.

With the increasing use of ultrasound (US) in evaluating facial muscles and vasculature [3, 4], Gossner proposed a special scanning method to visualize the nasal cartilage in 2014 [5]. This method involves placing the transducer in the axial plane with ample gel between the transducer's footprint and the nose tip/bridge. This technique is capable of visualizing the lateral and septal cartilages which appear as hypo‐ or an‐echoic owing to their composition of hyaline cartilage (which is rich in water, proteoglycans, and Type II collagen fibers) [6]. In other words, the homogeneous extracellular matrix allows sound waves to pass through with minimal reflection, resulting in a dark appearance on the US image.

Herewith, the aforementioned standard US method poses several challenges (Figure 1). The deeper portion of the septal cartilage is difficult to be visualized because the US beam runs parallel to the inner reflective plane of the cartilage, generating fewer echoes. Additionally, the mucosa of the internal nare is hard to be distinguished from the septal cartilage, due to the air‐filled vestibule impeding sound wave transmission. The superficial region of the nasal septum is usually thicker than the bottom part, especially at the nasal tip, hindering sound wave propagation to the deeper parts.

**FIGURE 1:**
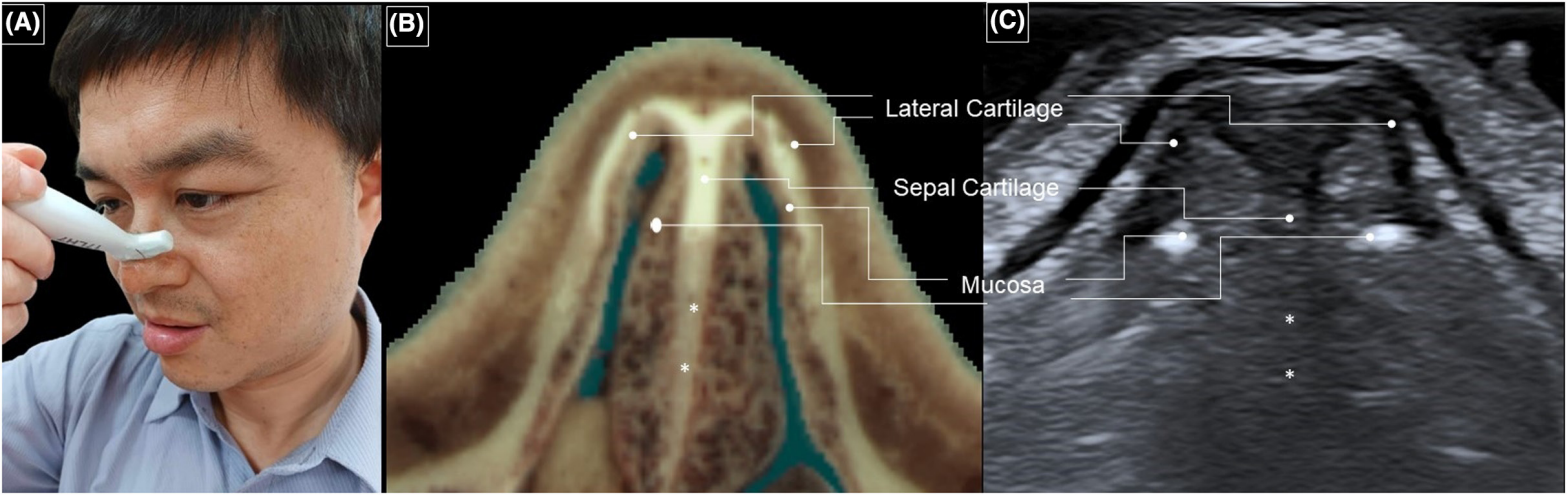
(A) The hockey‐stick transducer is positioned over the nasal bridge near the nasal tip in the horizontal plane. (B) The cadaveric cross‐sectional image and (C) the corresponding ultrasound scan show the nasal cartilage. Note that the bottom part of the nasal cartilage (*asterisk*) is barely visible in this maneuver. Cadaveric images are adapted from the Visible Human Project of the National Library of Medicine. Excerpts from these images, featured in the VH Dissector, are used with permission from Touch of Life Technologies Inc. Images showing the transducer position on our corresponding author's face are also used with permission for publication.

To overcome these challenges, we suggest that the transducer be rotated to the side of the nostril and compressing the nostril walls, expelling air and allowing sound waves to pass from the lateral nasal wall to the midline (Figure 2, Video S1). This method fully visualizes the entire septal cartilage from the nasal bridge to its bottom. Advantages of this approach include enhanced echo signal reflection (as the transducer's footprint becomes parallel to the septal cartilage) and accurate thickness measurement. While the soft tissue beside the nasal septum can be compressed (resulting in an underestimated measurement), the thickness of the septal cartilage remains unaffected. Needless to say, this would provide valuable preoperative insight as regards the septal cartilage thickness. By adopting this improved technique, surgeons can obtain clearer and more accurate images that might also aid in successful rhinoplasty and revision procedures alike. Nevertheless, when using this modified scanner maneuver, the physician should be aware that applying the transducer may cause some distortion of the surrounding tissues, leading to lateral displacement of the septal cartilage. This should be taken into account when interpreting the imaging of the region's anatomy.

**FIGURE 2:**
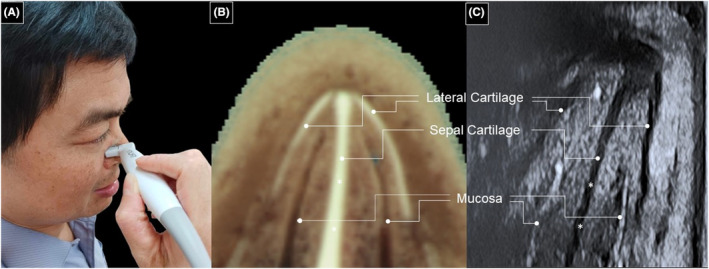
(A) The hockey‐stick transducer is rotated over the lateral wall of the nose, and pressure is exerted from the transducer's footprint to collapse the nasal vestibule. (B) The cadaveric cross‐sectional image and (C) the corresponding ultrasound scan show the nasal cartilage. Note that the bottom part of the nasal cartilage (*asterisk*) is clearly visible in this maneuver. Cadaveric images are adapted from the Visible Human Project of the National Library of Medicine. Excerpts from these images, featured in the VH Dissector, are used with permission from Touch of Life Technologies Inc. Images showing the transducer position on our corresponding author's face are also used with permission for publication.

## Conflicts of Interest

The authors declare no conflicts of interest.

## Supporting information


**VIDEO S1.** Dynamic ultrasonography for the nasal cartilage.

## Data Availability

The data that support the findings of this study are available from the corresponding author upon reasonable request.
